# Early childhood caries and its associated factors among 5-years-old Myanmar children

**DOI:** 10.3389/froh.2024.1278972

**Published:** 2024-01-25

**Authors:** Saw Nay Min, Duangporn Duangthip, Sherry Shiqian Gao, Palinee Detsomboonrat

**Affiliations:** ^1^ Postdoctoral Researcher Program in Dental Public Health, Faculty of Dentistry, Chulalongkorn University, Bangkok, Thailand; ^2^ Faculty of Dentistry, The University of Hong Kong, Hong Kong, Hong Kong SAR, China; ^3^ Department of Stomatology, School of Medicine, Xiamen University, Xiamen, China; ^4^ Department of Community Dentistry, Faculty of Dentistry, Chulalongkorn University, Bangkok, Thailand

**Keywords:** children, early childhood caries, Myanmar, prevalence, risk factors

## Abstract

**Introduction:**

Children's oral health plays a crucial role in their overall well-being and there is a significant gap in our understanding of early childhood caries (ECC) in Myanmar. This study aims to bridge this knowledge deficit by investigating the prevalence, causes, and potential interventions for ECC in the Myanmar population, providing crucial insights for future dental health policies and practices.

**Methods:**

Generally healthy 5-year-old kindergarten children from 7 districts in city were recruited. ECC was assessed through clinical examinations using decayed, missed, filled teeth (dmft). Additionally, demographic data of the children and their caregivers, along with information about the children's oral health-related behaviors, were gathered using a structured questionnaire.

**Results:**

Out of the 496 children, the overall prevalence of dental caries was 87.1% (mean dmft score: 5.57, SD: 4.6). Caries experience was categorized as severe (45.8%) and non-severe (41.3%). Decayed teeth constituted the major component of the dmft index (97.8%). Multiple logistic regression analysis revealed two significant factors associated with ECC prevalence: late toothbrushing initiation (OR: 2.54, *p* = 0.001) and dental visit experience (OR: 2.46, *p* = 0.010).

**Discussion:**

The study highlights the alarming ECC prevalence in 5-year-old children in Mandalay, Myanmar, with mostly untreated decayed teeth. The findings emphasize early preventive oral health measures for young children to reduce ECC burden in Myanmar.

## 1 Introduction

Early childhood caries (ECC), classified as a chronic non-communicable disease (NCD) by the WHO oral health resolution, is a prevalent oral ailment affecting children globally, with a pronounced impact on disadvantaged populations (1–3). Defined by the Bangkok Declaration on ECC, it is identified by the presence of one or more decayed (non-cavitated or cavitated lesions), missing or filled (due to caries) surfaces, in any primary tooth of a child under six years of age (4). ECC has a considerable impact on children due to its association with dental problems and general health issues (5). Like many NCDs, ECC arises not just from individual behavioral factors but also from a confluence of environmental, genetic, and societal influences (2, 6, 7). This multifactorial nature emphasizes the need to approach ECC not just as an isolated dental issue but as part of a broader spectrum of chronic diseases that are shaped by lifestyle, access to healthcare, and socio-economic conditions (7, 8). If left untreated, it can lead to inflammation, developmental challenges, dietary complications, social and psychological concerns, reduced academic performance (9, 10), and having decayed primary teeth is a significant indicator of future cavities in permanent teeth (11).

Recent systematic reviews reveal that the prevalence of ECC varies from 23%–90% across countries, making it a persistent challenge for the children population (12). While dental caries is generally well-controlled in countries with higher incomes, its prevalence is still on the rise in low and middle-income countries (13). Variations in the prevalence of ECC is more complex and exist within populations related to socioeconomic status, parents’ education, use of dental services and dietary habits (14–16). According to the systematic review conducted in Southeast Asia countries, children aged five to six years had a particularly high prevalence (25%–95%) of ECC (17). The Republic of the Union of Myanmar, a Southeast Asia country with a population of approximately 54 million people, is among the country where ECC is highly prevalent among young children (18–21). According to the National Oral Health survey (2016–2017), the mean dmft of 6-year-old-school children was 5.7 whereas the prevalence of untreated caries was 84.1% (20, 21). Another study also reported that 81.3% of 5–6 years old children had caries experiences with dmft 4.26 (22).

Despite Myanmar's National Health Plan (NHP) aiming for Universal Health Coverage (UHC) by 2030, insufficient personnel, language barriers, different cultures and beliefs, and inadequate transportation infrastructure remain obstacles to the provision of adequate health services to the whole country (23). In Myanmar, while there's a notable emphasis on five primary oral health initiatives, such as early childhood caries prevention, school-based programs, maternal education, fluoride accessibility, and oral cancer awareness, the provision of basic dental services in less accessible regions are still a hurdle. Moreover, the majority of dental professionals are more involved in advanced preventive measures than in primary care (18, 21–23). Even though accumulating oral health data is essential to implement national policies for oral health improvement, the oral health information system of Myanmar needs to be advanced in order to gather data on disease prevalence and trends (15, 18, 21, 22). For effective surveillance, WHO recommends that dental health surveys should be performed regularly every 5 years. So far, there is limited research on the updated prevalence and associated factors of ECC among Myanmar five-year-old children. This study aimed to investigate the ECC and its associated factors among five-year-old children in Mandalay city, Myanmar. The findings will also enhance our understanding of the underlying factors contributing to the high prevalence of ECC in Myanmar, which can have important implications for oral health policy and practice.

## 2 Materials and methods

### 2.1 Study design

This cross-sectional study was conducted in selected kindergartens in Mandalay city in Myanmar. The data collection was intended to be carried out from September to November 2021. The ethical approval was obtained from the Human Research Ethics Committee, Chulalongkorn University (HREC-DCU 2021-047). The study participants were 5 years-old children living in this Mandalay city. Mandalay, the second largest city, is situated in the middle part of Myanmar. Mandalay region has an estimated population of 1.22 million and the majority of the population is Burmese (24). The calculation for the minimum sample size was derived from a prior study (20) that indicated a prevalence rate of 84.1%. Given a 95% confidence interval and a 4% margin of error, it was determined that 385 participants would be necessary. Considering an expected participation rate of 80%, we needed to invite at least 481 children. The quota sampling following the proportion of the child population in each district was adopted. Five-year-old children from12 schools from the 7 districts were invited to the study and informed consent was obtained from the children's parents or guardians before implementation. The inclusion criteria included normal healthy cooperative Myanmar kindergarten children aged 60–71 months. The exclusion criteria were non-cooperative children, children with systemic diseases, and children with developmental delays and severe disabilities. Personal data of the children and their families were protected and confidential maintains ethical research practices.

### 2.2 Clinical examination and questionnaire

An oral examination was performed while the child was in a seated position on a chair under natural light. Dental caries was diagnosed based on visual and tactile criteria. Teeth were dried using sterile cotton rolls to improve the visibility of carious lesions. No radiographs were taken during the examination, and caries was recorded at the cavitation level only. The dental caries status of the children was assessed according to World Health Organization criteria (25) using a penlight, disposable dental mirror, and WHO-CPI probe. Caries experience in primary teeth was recorded in a modified oral health assessment form using dmft index as illustrated by WHO for epidemiological studies. A tooth was recorded as decayed (dt) when an unmistakable dentine carious cavity or when both a dentine carious lesion and a restoration were examined. A tooth was recorded as missing (mt) if it was extracted due to caries. A tooth was recorded as filled (ft) if a permanent filling without caries was present. The caries experience was classified as: caries-free (dmft = 0) and caries-positive (dmft > 0). The severity of dental caries was further categorized to: non-severe (dmft = 1–5), and severe (dmft > 6) based on the population being studied and the criteria used by researchers (26). When an individual's dmft score is between 1 and 5, it suggests a mild to moderate level of tooth decay. This category helps identify individuals who may need dental care but are not in an urgent or critical state. An individual with a dmft score greater than 6 falls into the severe category. This higher score indicates a significant level of tooth decay, multiple missing teeth, and/or numerous fillings. Such a condition might require more urgent dental interventions and possibly indicates a lack of access to dental care or other systemic health issues. Training and calibration exercises were conducted by two dentists to standardize the examination procedures before the study. A theoretical session using slide presentations and practical hands-on sessions on the 20 children in each survey were carried out. There was a high level of agreement (*k* > 0.80) between examiners for both inter-examiner and intra-examiner reliability. A structured questionnaire was used to gather information from the parent prior to the examination of each child's oral health condition. The following information was obtained: demographic data related to the parent (parental education and family household income), demographic data related to the child (child gender and age) and child oral health behaviors (frequency of snacks between meals, starting age for tooth brushing, frequency of tooth brushing, and dental visit experience, evaluating if the children have undergone dental treatment for caries or not). After data collection, researcher explained the educational materials provided to parents about ECC. This could include information on the causes and consequences of ECC, the importance of early dental hygiene, dietary advice, and the role of regular dental check-ups. This included referrals to dental clinics.

### 2.3 Data analysis

Data were analyzed by using the SPSS version 22.00 software (IBM Corp). Cohen's kappa coefficient was used to assess the intra- and inter-examiner reliability for caries diagnosis. Mann–Whitney *U* and Kruskal–Wallis tests were used to compare the mean dmft scores due to non-normally distributed data. A Chi-square test was performed to investigate the association between ECC and related factors. A multiple logistic regression analysis was performed to identify the variables that were statistically significant concerning ECC (0: caries-free, 1: having at least one caries). A backward stepwise procedure was used to eliminate any variables that were not statistically significant, and the final model was composed of only statistically significant factors. The Hosmer–Lemeshow goodness fit test was performed to evaluate the goodness-of-fit of the final model. Statistical significance was determined by a *p*-value of less than 0.05.

## 3 Result

A total of 532 five-year-old children from 12 schools in 7 districts were invited to participate in our study, with a response rate of 93.2% (*n* = 496/532). Thirty-six children were not included in the study due to not attending the schools on the examination day (*n* = 23), missing data (*n* = 8), and refusal of an oral examination (*n* = 5). The recruitment of the study children was shown in Supplementary Appendix 1. The study comprised 496 participants (258 girls and 238 boys) who received the oral examination and completed questionnaires.

The overall prevalence of dental caries was 87.1% and mean dmft score was 5.57 (4.6). Only 12.9% of all participant children were caries-free, the rest either had non-severe (41.3%) or severe (45.8%) caries experience. Decayed teeth were the main component for dmft (97.8%) with mean (dt = 5.45 ± 4.5) while the mean numbers of missing (0.08 ± 0.5) and filled teeth (0.04 ± 0.3) were very low. The distribution of dmft among the participants is presented in Table 1.

**Table 1:** Caries prevalence and dmft among participant children.

Variables	*N* (%)
Caries experience	
Caries-free	64 (12.9)
Non-severe	205 (41.3)
Severe	227 (45.8)
	Mean (SD)
Dmft	5.57 (4.6)
dt	5.45 (4.5)
ft	0.08 (0.5)
mt	0.04 (0.3)

dmft, decayed, missed, filled teeth; dt, decayed teeth; mt, missed teeth; ft, filled teeth.

Table 2 showed the demographic features and the association between ECC and its related factors in children. No significant differences in ECC prevalence and dmft score were found between girls and boys in this study. Concerning the socioeconomic factors, neither mother's education level nor the father's education level was significantly related to the caries status of the children. The prevalence of caries experiences and dmft scores of children with low family income were higher than those of children with moderate and high family income; however, the differences were not statistically significant. Regarding the children's oral health-related behaviors, children who had a sugary snack 2 times or less a day had a significantly lower caries prevalence and dmft score compared with those who had snacks more than 2 times (*p* = 0.046, *p *= 0.002). ECC prevalence and dmft scores were significantly lower in children who started tooth brushing ≤ 12 months (*p *< 0.001). In addition, children who had a dental visit experience had significantly higher ECC prevalence and dmft than children without a dental visit (*p* = 0.004, *p* < 0.001).

**Table 2:** Association between ECC and its related risk factors (*N* = 496).

Variables	*N*	ECC *N* (%)	*p*-value*	Mean dmft (±SD)	*p*-value
Child gender					
Girl	258	225 (87.2)	0.940	5.4 (4.5)	0.630
Boy	238	207 (86.9)		5.7 (4.7)	
Mother's education					
Middle secondary school or lower	145	130 (89.7)	0.454	5.8 (4.8)	0.730**
Secondary or High school	179	156 (87.2)		5.5 (4.3)	
Tertiary or University	172	(146) 84.9		5.3 (4.5)	
Father's education					
Middle secondary school or lower	132	117 (88.6)	0.601	5.7 (4.7)	0.818**
Secondary or High	170	147 (86.5)		5.6 (4.5)	
Tertiary or University	194	168 (86.6)		5.4 (4.5)	
Monthly household income in Kyat					
Low (<150,000)	44	39 (88.6)	0.672	6.2 (5.1)	0.580**
Middle (150,000–300,000)	290	255 (87.9)		5.6 (4.6)	
High (>300,000)	162	138 (85.2)		5.3 (4.3)	
Frequency of snacks per day					
2 times or less	276	233 (84.4)	0.046	4.9 (4.2)	0.002***
More than 2 times	220	199 (90.5)		6.3 (4.8)	
Starting age for tooth brushing					
≤12 months old	139	109 (78.4)	<0.001	3.9 (3.9)	<0.001***
More than 12 months old	357	323 (90.5)		6.2 (4.6)	
Frequency of tooth brushing					
2 times or more/day	113	97 (85.8)	0.650	5.3 (4.0)	0.451***
1 time/day	383	335 (87.5)		5.7 (4.7)	
Dental visit experience					
No	332	279 (84)	0.004	4.8 (4.3)	<0.001***
Yes	164	153 (93.3)		7.1 (4.7)	

* Chi-square test for caries prevalence.

** Kruskal–Wallis test.

*** Mann–Whitney *U*-test.

Table 3 showed the results of the final logistics regression model of significant factors with ECC (dmft > 0). Based on bivariate analysis, three variables were selected for multiple regression analysis: frequency of snacks per day, the age at which tooth brushing began, and experience with dental visits. After adjusting the confounding factors, only two factors—starting age for toothbrushing and dental visit experiences were significantly associated with the prevalence of ECC. It was found that caries experiences were significantly higher in children who began brushing their teeth after 12 months of age (OR: 2.54, *p *= 0.001) and those with prior dental visit experiences (OR: 2.46, *p* = 0.010). The Hosmer and Lemeshow result indicated a good logistic regression model fit with *p* value greater than 0.05 (*p* = 0.520).

**Table 3:** Multiple logistic regression of factors associated with ECC prevalence.

Variables	Odds ratio	95% CI	Wald (χ^2^)	*p*-value
Starting age for tooth brushing				
12 months old^a^				
More than 12 months old	2.54	1.472–4.374	11.239	0.001
Dental visit experience				
No^a^				
Yes	2.46	1.238–4.888	12.889	0.010

Statistically significant *p* < 0.05.

^a^ Reference group, CI, Confidence interval; Wald (χ^2^), chi-square value.

## 4 Discussion

The finding of the present study demonstrated the overall prevalence of dental caries was 87.1% (mean dmft score: 5.57, SD:4.6) and decayed teeth constituted the major component of the dmft index (97.8%). Despite the recent developments in Myanmar's oral health, only a few data exist regarding ECC in young children. The results of this study provide the updated prevalence of ECC and associated factors among five-year-old children in Mandalay, Myanmar, which could be used to develop oral health prevention and promotion strategies for this age group. The results of this study showed similar caries and dmft prevalence rates to those reported in the Myanmar National Oral Health Survey (84.1% and 5.71) (20, 21) and in other studies conducted in different cities including in Naypyidaw (81.3%, 4.26 ± 3.76) (22) and Yangon (84.1%, 5.84 ± 4.57) (19). These findings demonstrated that ECC prevalence remains high and severe among young Myanmar children. Furthermore, an overview report of Myanmar's oral health situation revealed that the prevalence and experience of caries among five-year-old children has remained relatively unchanged since the 2006 Pathfinder Survey (18, 20).

In Southeast Asia, preschool children in Myanmar, the Philippines, Lao PDR, and Cambodia experience very high rates of Early Childhood Caries (ECC), with prevalence surpassing 85%. In contrast, Brunei and Singapore show notably lower rates of ECC among preschoolers, at 59% and 49% respectively (17). Across different continents, the prevalence of ECC varies significantly. In Asia, most studies indicate that over half of the children suffer from dental caries. In South America, around two-thirds of studies report a prevalence exceeding 50%. All studies from Africa and the Middle East show a similar or higher prevalence. However, in Europe, the majority of studies suggest a lower prevalence of ECC compared to other continents (27).

The study underscores the enduring high caries prevalence in Myanmar, with a low percentage of filled teeth, indicating barriers to oral healthcare access, such as financial constraints and limited awareness or resources. It also suggests that children who visit dental clinics have higher caries rates, likely seeking treatment for pain rather than preventive care. It is a similar finding to that of previous studies (28, 29). This calls for increased awareness and access to regular dental care. Globally, policies addressing ECC vary, including Medicaid and the Children's Health Insurance Program in the USA, Hong Kong's Oral Health Education Unit, and Thailand's National Health Act. These policies focus on health promotion, fluoridation, and sugar taxation to combat ECC (30).

The present study found that children who had not initiated tooth brushing by one year of age had increased caries risk, similar to findings from other studies (29, 31). According to the American Academy of Pediatric Dentistry (AAPD), it is recommended that toothbrushing should begin when the first teeth of a child erupt (32). However, the frequency of daily brushing was not related to the risk of ECC in this study which had been found in other studies (10, 28, 31). A number of factors, including improper brushing technique, brushing duration, and the brushing with fluoride toothpaste on early childhood caries (ECC) which were not estimated in this study, may have contributed to the results of this study. Nonetheless, parents should be encouraged to start brushing their children's teeth as soon as the first teeth erupt. Although no significant association was found between the frequency of snacks and caries experiences in the final logistic regression model, children with daily snacking more than two times presented more dmft scores compared to those with daily snacking two times or less. The result was in line with previous studies (16, 33). Sugary snacks and drinks are easily available from snack shops as well as from the school canteen in Myanmar. Therefore, these findings highlight the need for more comprehensive policies and regulations to control the availability of sugary snacks and drinks in schools and to provide more information about the negative health effects of consuming sugary snacks and drinks to students and parents.

It is widely acknowledged that socioeconomic status is an important risk indicator for ECC (34). Previous studies (9, 29, 34), demonstrated that a lower parental education level results in higher caries prevalence amongst participants. However, the present study found no significant association between parental education and ECC prevalence, though children with lower parental education levels had a higher prevalence. This finding was similar to the prior studies (14, 19, 35), and the potential explanation for the conflicting results concerning parental education across various studies may be attributed to the fact that the majority of studied parents were highly educated. This study also found no significant association between family household incomes and ECC prevalence. It is possible that other factors such as oral health knowledge and dietary habits, and the current socio-economic environment influenced this finding. Strategies to prevent ECC should focus on improving access to preventive oral care and providing education to the parents. Furthermore, population-approaches, including public education, public policy implementation, healthy behavior promotion and risk factor reduction, as well as high-risk approaches targeting those individuals most likely to develop ECC (e.g., those from disadvantaged backgrounds or with existing dental caries), such as early detection, efficient treatment, and targeted interventions, should be used in a complementary manner to reduce the burden of ECC.

The present study has some limitations with regard to the determination of causal relationships between associated factors and the results, due to its cross-sectional design. Moreover, this is our limitation in collecting various factors, which may not be comprehensive, and we did not gather the full context of the family, parents, caregivers, and infant care practices. Due to the instability politics under the military coup in 2021, random sampling could not be performed as planned. Thus, quota sampling, which is a non-probability sampling, was instead adopted to ensure the safety of the researchers and participants. Thus, the results of this survey may possibly contain sampling bias. Furthermore, the evaluation of the caregivers' responses in terms of reliability was not conducted. It's worth noting that recall bias might have impacted the caregivers' answers. Additionally, focusing exclusively on preschool children residing in Mandalay city could potentially restrict the applicability of the research findings to the entire nation. Despite this, the study was able to update the current status of ECC in Myanmar especially in the midst of severe political instability and economic crisis. To more thoroughly investigate the determinants of ECC, further cohort research with the representative of the population is needed. A well-designed national study using representative samples from all cities in Myanmar must be conducted to confirm the results reported in this study.

## 5 Conclusion

Early Childhood Caries (ECC) is highly prevalent in Mandalay city, Myanmar. Almost all decayed primary teeth are untreated. ECC is significantly associated with the starting age for toothbrushing and the history of previous dental visits in this study. National oral health policies and programs should be revisited to reduce the burden of ECC and improve child oral health in Myanmar.

## Data Availability

The original contributions presented in the study are included in the article/Supplementary Material, further inquiries can be directed to the corresponding authors.
