# Supplementary figures and images for: Early childhood caries and its associated factors among 5-years-old Myanmar children
Source: Front Oral Health. 2024 Jan 25;5:1278972. doi: 10.3389/froh.2024.1278972 (PMC10850244; doi:10.3389/froh.2024.1278972)

### *Appendix 1*

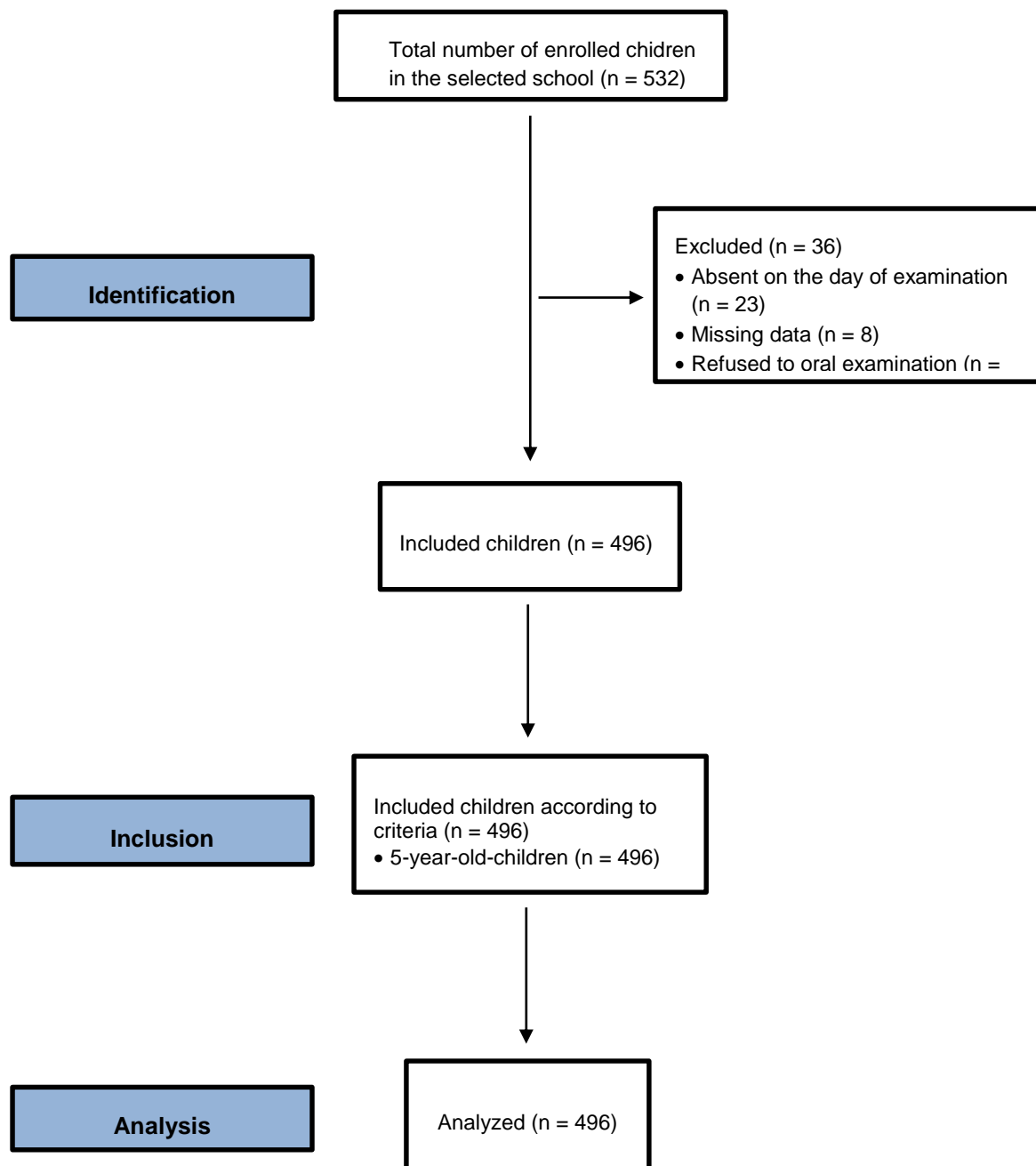

**Supplementary Figure 1.** Flow diagram of recruitment of children

Supplement: Supplementary file 1 [file Image1.pdf]
